# Intranasal gene therapy to prevent infection by SARS-CoV-2 variants

**DOI:** 10.1371/journal.ppat.1009544

**Published:** 2021-07-15

**Authors:** Joshua J. Sims, Jenny A. Greig, Kristofer T. Michalson, Sharon Lian, R. Alexander Martino, Rosemary Meggersee, Kevin B. Turner, Kalyani Nambiar, Cecilia Dyer, Christian Hinderer, Makoto Horiuchi, Hanying Yan, Xin Huang, Shu-Jen Chen, James M. Wilson

**Affiliations:** Gene Therapy Program, Department of Medicine, Perelman School of Medicine, University of Pennsylvania, Philadelphia, Pennsylvania, United States of America; University of Michigan, UNITED STATES

## Abstract

SARS-CoV-2 variants have emerged with enhanced pathogenicity and transmissibility, and escape from pre-existing immunity, suggesting first-generation vaccines and monoclonal antibodies may now be less effective. Here we present an approach for preventing clinical sequelae and the spread of SARS-CoV-2 variants. First, we affinity matured an angiotensin-converting enzyme 2 (ACE2) decoy protein, achieving 1000-fold binding improvements that extend across a wide range of SARS-CoV-2 variants and distantly related, ACE2-dependent coronaviruses. Next, we demonstrated the expression of this decoy in proximal airway when delivered via intranasal administration of an AAV vector. This intervention significantly diminished clinical and pathologic consequences of SARS-CoV-2 challenge in a mouse model and achieved therapeutic levels of decoy expression at the surface of proximal airways when delivered intranasally to nonhuman primates. Importantly, this long-lasting, passive protection approach is applicable in vulnerable populations such as the elderly and immune-compromised that do not respond well to traditional vaccination. This approach could be useful in combating COVID-19 surges caused by SARS-CoV-2 variants and should be considered as a countermeasure to future pandemics caused by one of the many pre-emergent, ACE2-dependent CoVs that are poised for zoonosis.

## Author summary

During the COVID-19 pandemic, variants of SARS-CoV-2 have emerged that are more deadly, more transmissible, and that evade immune response. These variants threaten to reduce the efficacy of first-generation vaccine and monoclonal antibody therapeutics. An additional problem with the existing vaccine strategy is that immune-compromised patients do not respond to active vaccinations. To address these issues, we developed a gene therapy-based strategy to prevent COVID-19 using a viral vector delivered by nasal spray. Unlike vaccines, this passive prophylaxis does not require that the patient mount an immune response. Instead, airway cells are engineered to secrete an antiviral decoy protein that can intercept viral particles before they cause infection. This decoy is based on human ACE2, the natural receptor for SARS-CoV-2. This design imparts resistance to SARS-CoV-2 viral evolution: viral mutations that evade the ACE2 decoy are unlikely to emerge because they would also render the virus less capable of infecting cells and therefore be less fit. Indeed, our ACE2 decoy neutralizes all SARS-CoV-2 variants that we tested and even binds distantly related coronaviruses that have not yet crossed over from animals to humans. This suggests that the ACE2 decoy gene therapy approach could be effectively deployed to vulnerable populations in the COVID-19 pandemic and potentially to broader populations at the outset of future coronavirus pandemics.

## Introduction

The COVID-19 pandemic resulted in more than 3.5 million deaths worldwide in the 18 months that followed the first known case. Highly effective vaccines are dramatically slowing transmission and deaths in some countries. However, vulnerable populations of immune-compromised patients exist that will not respond to vaccination. Additionally, variants of the etiologic agent, SARS-CoV-2, have emerged with the potential to circumvent antibody neutralization, suggesting that first generation monoclonal therapeutics and vaccines may eventually become less effective. Evidence suggests these two critical issues may be linked, in that long-term infection of immune-compromised patients may serve to accelerate viral evolution [1].

We set out to address these issues by developing a gene therapy-based, passive prophylaxis to SARS-CoV-2 infection using an antiviral decoy transgene that may be especially resistant to viral evolution. ACE2 decoys—soluble forms of angiotensin-converting enzyme 2—are considered a protein therapeutic in the treatment of COVID-19 patients [2,3]. We isolated an affinity-matured ACE2 decoy that broadly neutralizes SARS-CoV-2 variants and demonstrate its potential for preventing COVID-19 when expressed from an adeno-associated virus (AAV) following intranasal (IN) delivery. We have reported previously on the effectiveness of IN AAV to express antibodies that broadly neutralize pandemic strains of influenza [4–7]. We report a novel AAV capsid with superior gene transfer in the primate proximal airway and demonstrate therapeutic levels of active protein expression following AAV-decoy treatment. We also discuss the applications of the IN-AAV decoy approach for current and future coronavirus pandemics.

## Results

### ACE2 decoy affinity maturation enhances neutralization of SARS-CoV-2 300-fold

We initially constructed a decoy receptor by fusing a human ACE2 fragment to the human IgG4 Fc domain. We cloned this first-generation decoy into AAV and delivered it as a nasal spray into nonhuman primates (NHPs). Although we detected decoy expression in nasal lavage fluid (NLF), the decoy was not produced at levels sufficient to overcome the low neutralizing potency of this protein (S1 Fig). We therefore set out to affinity-mature the ACE2 protein sequence.

**Fig 1:**
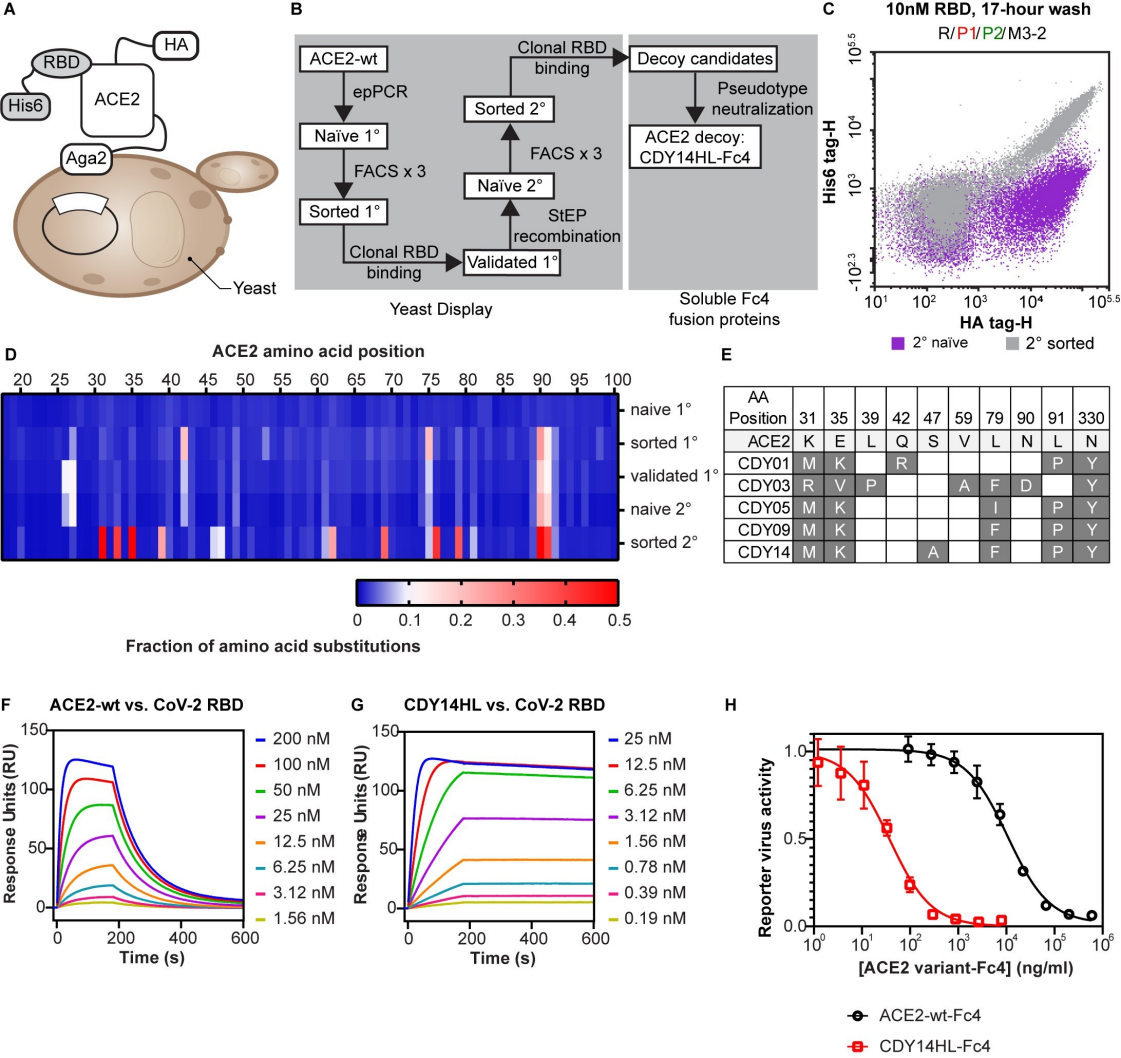
ACE2 Decoy Receptor Engineering. **A.** Yeast display (YD) system **B**. Decoy affinity maturation and candidate selection process. **C**. Flow cytometry analysis of the naïve (purple) and sorted populations (gray) from the secondary YD library. **D**. NGS analysis of plasmid populations recovered from rounds of YD. **E**. Mutations accumulated in the top five decoy-Fc4 candidates. **F and G**. SPR binding analysis for CoV-2 RBD injected over surface-immobilized ACE2-wt (F) or CDY14HL (G). **H**. Wuhan CoV-2-Pseudotyped lentiviral reporter neutralization assay of ACE2-wt-Fc4, and CDY14HL-Fc4. Data for at least three independent measurements are presented as average ± standard error of the mean.

We generated diverse (>10^8^ transformants) ACE2 variant libraries in a yeast-display format[8] using error-prone polymerase chain reaction (PCR; Figs 1A and S2). We screened the primary libraries in rounds of fluorescence-activated cell sorting (FACS; Fig 1B). We selected populations with better binding to SARS-CoV-2 receptor binding domain (RBD) and tracked library convergence with deep sequencing (Fig 1C and 1D). Frequently observed mutations from our primary library sorts overlap partially with mutations reported by others [3,9], including substitutions at T27 and N90 glycan disruption (S2 Fig). Validated clones from the sorted primary libraries (S2 and S3 Figs) seeded a secondary library formed by mutagenic recombination[10,11], which we screened using stringent off-rate sorting[12] (Fig 1C).

We expressed ACE2 variants from several stages of the yeast-display screening as soluble IgG4 Fc fusions, evaluated expression titers, and predicted IC_50_ for SARS-CoV-2 neutralization using reporter virus (S3 Fig). The most potent neutralizing variants converged upon similar substitutions at five positions: 31, 35, 79, 330, and N90 glycan disruption (Fig 1E). After further characterization, we selected CDY14-Fc4 as the most improved ACE2 decoy variant. To avoid off-target effects *in vivo*, we ablated ACE2 enzyme activity by introducing H345L[13] at no cost to potency (S3 Fig). By surface plasmon resonance (SPR) the active site-null CDY14HL-Fc4 bound SARS-CoV-2 RBD with 1,000-fold improved affinity (29 nM for wtACE2-Fc4 vs. 31 pM for CDY14HL-Fc4; see Fig 1F and 1G). CDY14HL-Fc4 neutralized Wuhan-Hu-1 SARS-CoV-2 reporter nearly 300-fold better than the un-engineered ACE2 decoy (IC_50_ 37 ng/ml for CDY14HL-Fc4 vs. 11 μg/ml for ACE2-wt-Fc4; see Fig 1H).

### ACE2 decoy is effective against SARS-CoV-2 variants and SARS-CoV-1

**Fig 2:**
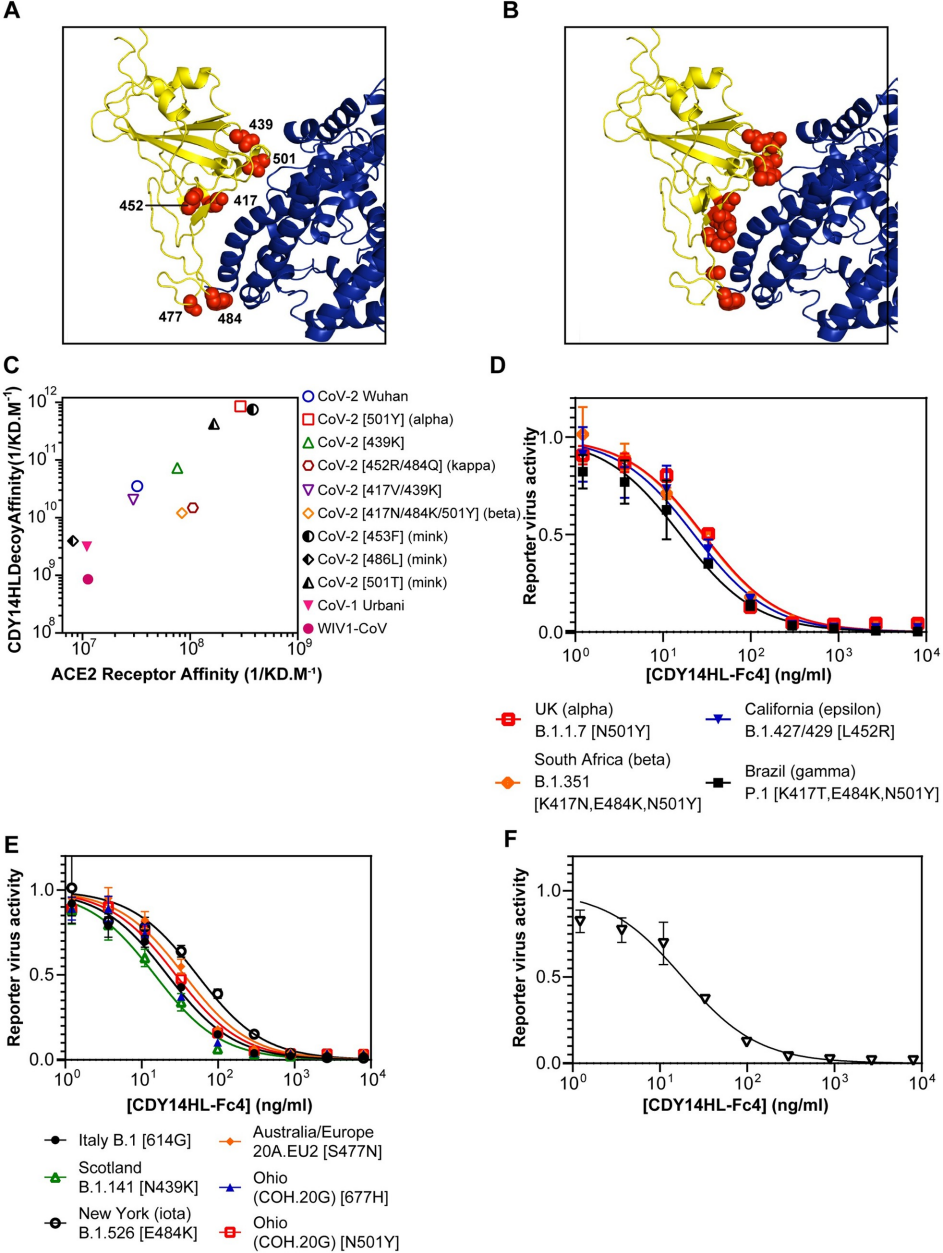
ACE2 Decoy Binding and Neutralization Across Diverse CoVs. **A.** Structural model (7DF4.pdb [42]) of Wuhan CoV-2 RBD (yellow) bound to human ACE2 (blue) RBD. The six RBD residues frequently mutated in emerging CoV-2 variants are shown in red spheres. **B**. Wuhan CoV-2 model as in (A) but with RBD residues highlighted at the ACE2 interface (red spheres) which differ from CoV-1. **C**. SPR measurements: ACE2-wt-Fc4 or CDY14HL-Fc4 binding to various purified recombinant RBD proteins. **D**. CDY14HL-Fc4 titrations using pseudotyped lentivirus reporters encoding CoV-2 spike proteins from four US-CDC Variants of Concern. RBD mutations are indicated in brackets, however in many cases other spike mutations exist that are not listed. **E**. Pseudotype titrations for six additional CoV-2 spike variants. **G**. Pseudotype titration for CoV-1 spike reporter virus. Reporter virus activity data are presented as mean ± standard error of the mean for at least three replicate titrations.

Unlike antibodies, decoy inhibitors may achieve broad neutralization and escape mutant resistance; decoy escape would require a viral mutation likely to reduce binding to the endogenous receptor and thus overall fitness. By the same logic, decoys should maintain neutralizing potency even as viruses evolve tighter binding to their receptor to achieve more efficient infection. Mutations at six sites at the RBD:ACE2 interface have arisen in multiple independent SARS-CoV-2 lineages (Fig 2A). To varying degrees, mutations at these sites have been reported to confer enhanced ACE2 binding, transmissibility, virulence, and escape from first-generation monoclonal antibody therapeutics or even active vaccines[14]. To assess the potential of our decoy for broad neutralization, we measured ACE2-Fc4 (a surrogate for the native receptor) and CDY14HL-Fc4 (the therapeutic decoy) affinities across a diverse panel of CoV RBDs using SPR. We chose RBDs that contain mutation combinations observed at these six sites (Fig 2A), as well as other strains that have been reported. These include strains under positive selection in the course of the 2020 pandemic (e.g., 439K from B.1.141, first isolated in Scotland [15]; 501Y from B.1.1.7/alpha variant, first isolated in the UK; 417N/484K/501Y from B1.351/beta variant, first isolated in the Republic of South Africa [16]; 452R/484Q from B.1.617.1/kappa variant, first isolated in India), and mink-adapted isolates[17]. Several emerging SARS-CoV-2 variants with improved affinity for ACE2-Fc4 (501Y, 453F, and 501T) also bind CDY14HL-Fc4 more tightly (Figs 2B and S4), while 417N/484K/501Y, 439K, 439K/417V, and 452R/484Q only modestly alter binding to ACE2-Fc4 or CDY14HL-Fc4. While 486L reduces affinity for CDY14HL-Fc4, it does so for ACE2-Fc4 proportionally.

Next we examined RBDs from more distantly related CoVs. Comparative structural analysis indicates that SARS-CoV-1 and SARS-CoV-2 differ at 11 of the 19 interfacial residues critical for ACE2-binding (Fig 2B) [18]. A similar degree of divergence at the ACE2 binding site exists for the pre-emergent bat coronavirus, WIV1-CoV[19]. Remarkably, the decoy binds both of these distantly related CoV domains with higher affinity than the endogenous ACE2 receptor (Fig 2C). The tight coupling of decoy and receptor affinities seen in the panel of CoV-2 variants is extended across the clade of ACE2-dependent betacoronaviruses and underscores the potential for broad neutralization by CDY14HL-Fc4.

Next, we compared decoy neutralization across diverse SARS-CoVs using pseudotyped lentivirus reporters. We observed potent neutralization of pseudotypes for the 4 strains (alpha, beta, epsilon, and gamma) currently considered Variants of Concern by the United States Centers for Disease Control (Fig 2D). The neutralization IC50s are lower for these strains than for the Wuhan pseudotype, reaching 16 ng/ml for the P.1/gamma variant reporter (Table 1). This increased potency may be explained by a combination of effects, including the RBD-exposing 614G mutation [20,21] that occurs in the later lineages (Fig 2D and Table 1), and the decoy affinity enhancement associated with some RBD substitutions contained within these strains (Fig 2C). We measured neutralizing potencies for 6 additional pseudotypes encoding spike variants that have emerged on different continents and at different stages of the COVID-19 pandemic (Fig 2E). Most are neutralized with IC50 values under 30 ng/ml (Table 1). Remarkably, selecting for increased binding to the SARS-CoV-2 RBD resulted in very potent neutralization of the phylogenetically distinct SARS-CoV-1 reporter virus (IC_50_ = 18 ng/ml, Fig 2F and Table 1). Taken together with the binding survey, these data indicate that structural features of the ACE2 interface have been retained through the stages of directed evolution. Moreover, the data predict that CDY14HL-Fc4 could protect against current, emerging, and future pandemic ACE2-dependent CoVs.

**Table 1:** Potency of CDY14HL-Fc4 in the neutralization of lentiviruses pseudotyped with CoV spike variants.

1^st^ Seq Location/ Common Name	PANGO lineage and other lineage names	RBD mutations	614	IC50 mean ± SEM (ng/ml)
Wuhan	A.1 (Nextstrain 19B)	-	D	37 ± 6
614G	B.1	-	G	22 ± 3
Scotland/EU	B.1.141	N439K	G	15 ± 3
UK	B.1.1.7/alpha	N501Y	G	29 ± 4
South Africa	B.1.351/beta	K417N,E484K,N501Y	G	28 ± 5
Brazil	P.1/gamma	K417T,E484K,N501Y	G	16 ± 5
Ohio-501Y	(COH.20G/501Y)	501Y	G	22 ± 3
Ohio-677H	(COH.20G/677H)	-	G	28 ± 4
California	B.1.427/B.1.429/epsilon	L452R	G	23 ± 6
Australia/Europe	(20A.EU2)	S477N	G	36 ± 5
New York	B.1.526/iota	E484K	G	53 ± 17
CoV1-Urbani	N/A	N/A	N/A	18 ± 5

### ACE2 decoy diminishes SARS-CoV-2 sequelae in transgenic mice

We considered SARS-CoV-2 challenge studies in hamsters, macaques, and the hACE2 transgenic (TG) mice to evaluate the *in vivo* efficacy of an AAV vector expressing CDY14HL-Fc4. In all models, achieving evidence of viral replication *in vivo* requires virus doses that far exceed those required for human transmission. Furthermore, the clinical and pathologic sequelae of SARS-CoV-2 exposure is attenuated in these species compared to severely affected humans. The most significant limitation, however, is that all the challenge models require direct pathogen delivery to the lung in order to demonstrate pathology, which does not simulate the mechanism of the AAV decoy product, which focuses on localized expression in the proximal airway following intranasal delivery to reduce SARS-CoV-2 infection and its consequences. We therefore selected the hACE2 TG mouse model for three reasons: 1) we can characterize disease by measuring viral loads, clinical sequelae, and histopathology; 2) we can use an IN route of administration as we would in humans, realizing this deposits vector in the proximal and distal airways of the mouse, while IN delivery in humans is restricted to the proximal airway; and 3) we can leverage the extensive experience of murine models in de-risking human studies of AAV gene transfer.

**Fig 3:**
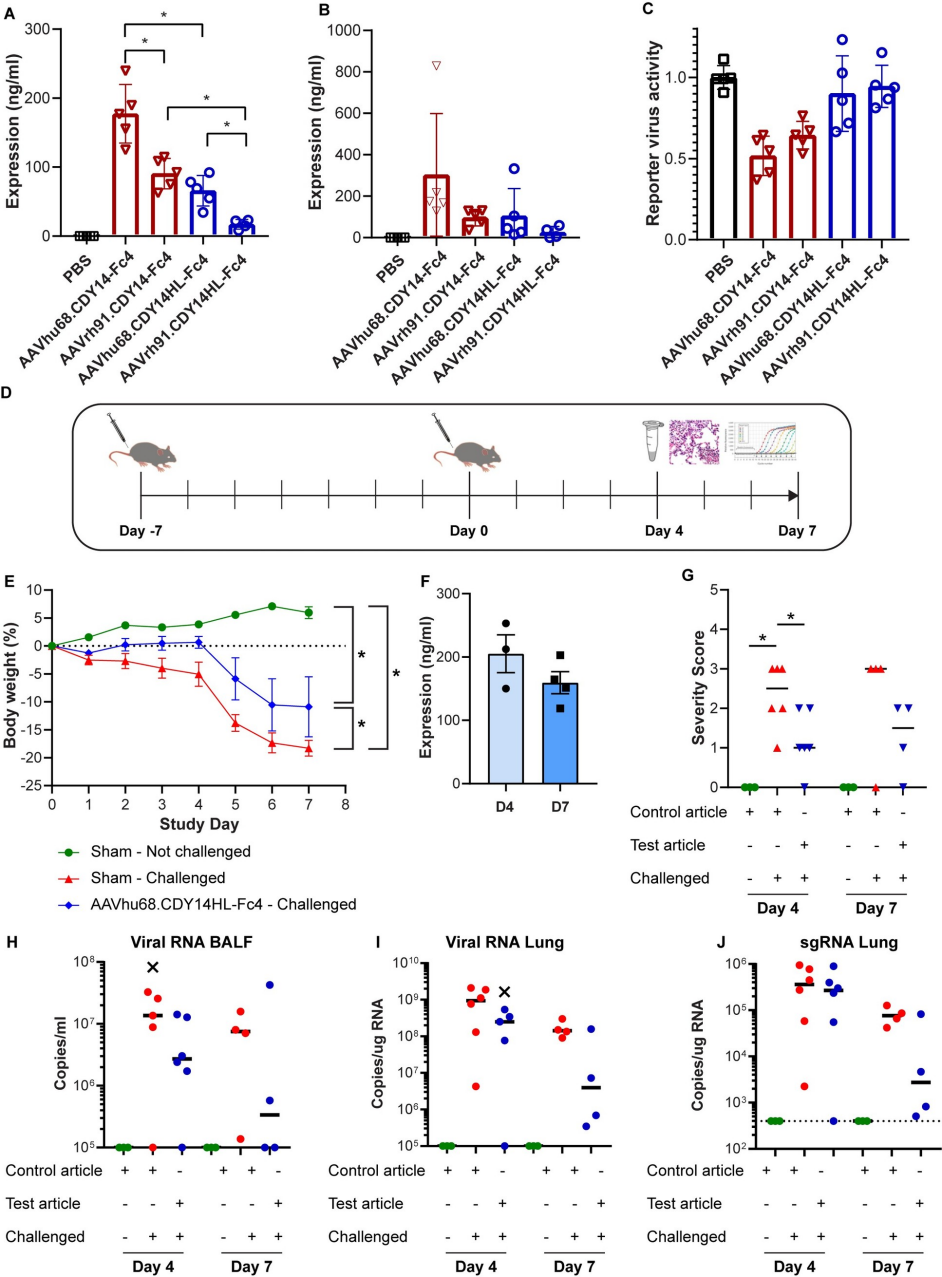
Protection in the human ACE2 transgenic mouse model. BAL from vector-treated animals analyzed for: **A.** decoy protein by MS; **B.** SARS-CoV-2 spike ELISA; **C.** neutralization of SARS-CoV-2 pseudotyped lentivirus. **D.** Challenge study design. **E.** Weight loss in the animals that were sustained for 7 days; one animal in the vehicle and vector treated groups required euthanasia. **F**. MS assay of expression in ASF (corrected for BAL dilution). **G.** Pulmonary inflammation histopathology scores of tissues harvested at days 4 and 7. **H.** Viral RNA in BAL. **I.** Viral RNA in lung. **J.** Sub-genomic RNA in lung. Outliers are indicated with X.

We conducted pilot studies in wild-type mice to determine which decoy protein (CDY14-Fc4 vs. CDY14HL-Fc4) and capsid (clade F AAVhu68 vs. clade A AAVrh91) maximized expression following *in vivo* gene delivery. We administered 10^11^ genome copies (GC) of vector (S6H Fig) and recovered broncho-alveolar lavage (BAL) samples 7 days later to evaluate ACE2 decoy protein expression and activity (Fig 3A–3C). Based on mass spectrometry (MS) protein measurements, the AAVhu68 capsid was more efficient than the AAVrh91 capsid in transducing mouse lung. The HL mutation modestly reduced expression (p<0.007). Importantly, we found a direct correlation between decoy expression levels and the ability to bind to SARS-CoV-2 spike protein and neutralize a SARS-CoV-2 pseudotype, demonstrating function of the decoy expressed from airway tissues (Fig 3A–3C). We selected CDY14HL-Fc4 as the clinical candidate transgene and the AAVhu68 capsid for the mouse challenge studies (Fig 3D).

We IN delivered AAVhu68-CDY14HL-Fc4 or vehicle to hACE2-TG mice. Seven days later, animals were challenged with SARS-CoV-2 (280 pfu), followed clinically (observation and daily weights), and necropsied on days 4 and 7 after challenge for tissue and BAL analysis (Fig 3D). Expression of CDY14HL-Fc4 in BAL normalized for dilution was in the range of the IC_50_ measured *in vitro* and in the pilot studies (Fig 3F). Sham-treated SARS-CoV-2 challenged animals demonstrated statistically significant weight loss as has been described by others[22–24]. We observed significantly less weight loss amongst vector-treated animals (which we followed for 7 days) compared to untreated animals (observed on days 4 and 7; p<0.05, linear mixed effect modeling). The vector-treated animals also significantly differed from the untreated, unchallenged animals (Fig 3E). Interestingly, the clinical outcome of the treatment was better among females than males, although we noted significant variations within the treated group (S5 Fig).

Histopathology of the lungs from vehicle-treated animals challenged with SARS-CoV-2 revealed findings similar to that previously described in this model[24]. As expected, tissues from animals not challenged with SARS-CoV-2 demonstrated no histopathology. Samples from days 4 and 7 showed reduced lung pathology in AAVhu68.CDY14HL-Fc4 treated animals vs. the vehicle-treated animals; the day-4 samples achieved statistical significance (p<0.05; Wilcoxon Rank Sum Test). (Fig 3G). Compared to vehicle-treated animals, viral RNA in BAL and lung homogenate was diminished at day 4 and 7 in AAVhu68.CDY14HL-Fc4 treated animals (Fig 3H and 3I). The greatest reductions were at day 7 for both BAL (26-fold) and lung tissue (35-fold). Impact of the AAVhu68.CDY14HL-Fc4 on SARS-CoV-2 replication, as determined by median sgRNA levels, was greatest at day 7 (27-fold reduction, Fig 3J). Although there was substantial inter-animal variation, 2/5 animals in the treated groups showed nearly complete abrogation of viral replication and little weight loss by day 7.

### AAV delivery yields therapeutic ACE2 decoy levels in nonhuman primates

**Fig 4:**
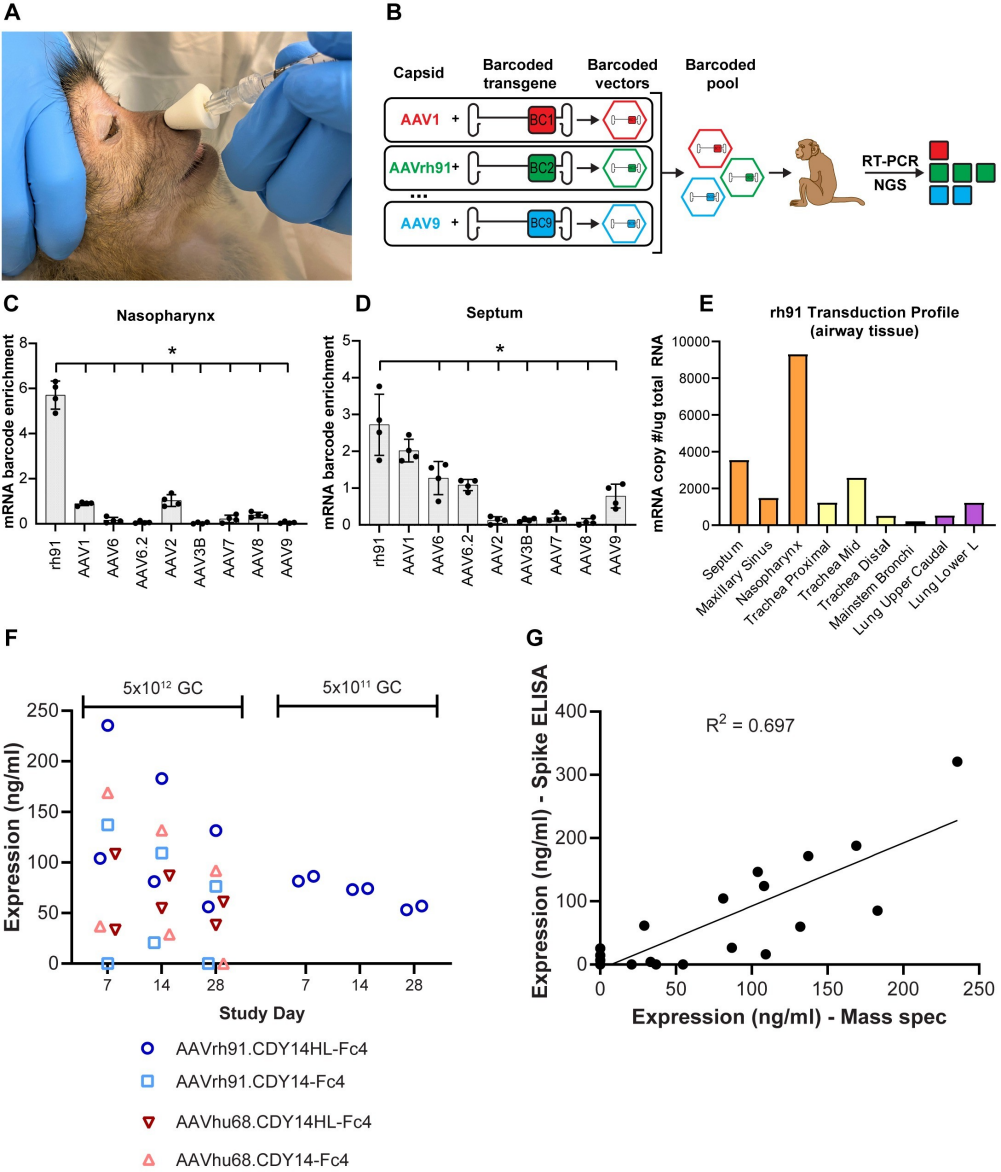
AAV-Delivered Decoy Expression in the Airway of NHP. **A.** NHP vector delivery via MAD Nasal. **B**. Pooled capsid comparison using mRNA barcoding. mRNA barcode enrichment scores (average across 4 barcodes ± SD) in (**C**) nasopharynx and (**D**) septum. **E.** AAVrh91 transduction profile in airway barcode study. Absolute rh91 transduction values calculated from the barcode study are shown for proximal airway (yellow), large conducting airway (orange) and distal lung (purple). No rh91 transcripts detected for nasal vestibule, oropharynx tonsil, tongue, lung (upper cranial, middle, and lower right). **F**. MS assay of expression in ASF (corrected for BAL dilution) for NHPs (2/group) dosed with ACE2 decoy vector. **G.** Correlation between decoy protein by MS and spike binding by ELISA. Data includes d7 and d14 samples from (F) plus NLF of 3 naïve macaques. We excluded one naïve animal from ELISA analysis because of background binding presumably due to a prior coronavirus infection.

Next, we determined which AAV capsid was most efficient at transducing cells of the nonhuman primate (NHP) proximal airways—the desired cellular targets for COVID-19 prophylaxis following nasal delivery of vector. We administered vector using a previously approved intranasal mucosal atomization device (MAD Nasal), which comprises an atomizing tip with a soft conical nostril seal fit on a standard syringe (Fig 4A). A mixture of 9 AAV serotypes with uniquely barcoded transgenes were administered via the MAD Nasal to an NHP. Tissues were harvested 14 days later for evaluation of relative transgene expression using the mRNA bar-coding technique (Fig 4B)[25]. The novel Clade A capsid (AAVrh91) we isolated from macaque liver performed best in the nasopharynx and septum (Fig 4C and 4D) with low but detectable expression levels in large airways and distal lung (S6A–S6G Fig). Clade E and F capsids performed better than AAVrh91 in some non-target tissues such as distal lung (S6A–S6G Fig). The profile of expression from AAVrh91 illustrates relative distribution of transgene expression with proximal airway structures>intra-pulmonary conducting airway>distal lung (Fig 4E).

To determine the candidate for clinical evaluation, we conducted a final NHP study where groups of 2 animals were administered 5x10^12^ GC of vectors that differed with respect to capsid (AAVhu68 vs. AAVrh91) and transgene cassettes (CDY14-Fc4 vs CDY14HL-Fc4). Nasal lavage fluid (NLF) samples were harvested on days 7, 14, and 28, and animals were necropsied on day 28 for biodistribution. Analysis of pulmonary tissues from day 28 revealed broad distribution throughout the proximal and distal airway, with AAVrh91 demonstrating superior gene transfer to proximal airway structures, as suggested by the barcode study (S6H and S6I Fig). We estimated decoy protein concentrations in the air surface fluid (ASF) based on dilution-adjusted MS measurements of NLF (Fig 4F). The effective concentrations at the ASF were in the range that demonstrated neutralization in the *in vitro* assay. Expression was slightly higher with AAVrh91 vs. AAVhu68. Unlike the mouse pilot study, we saw no obvious expression defect with the addition of the ACE2-disabling HL mutation in the primate airway, though the groups’ size in the NHP study was too low for a statistical comparison. A subset of samples evaluated for binding to the spike protein of SARS-CoV-2 showed a good correlation with decoy protein as measured by MS. This indicates that the decoy protein produced *in vivo* in proximal airways was indeed functional (Fig 4G).

Based on these data, we selected a candidate for subsequent clinical evaluation called GTP404. This candidate utilizes AAVrh91 as the capsid because of its transduction profile and CDY14HL-Fc4 as the transgene because it retained broad and potent neutralizing activity in the setting of an ACE2-disabling mutation that may improve safety, particularly for long-term expression of the decoy. In preparation for Investigational New Drug- (IND-) enabling studies, we administered GTP404 at a 10-fold lower dose to two additional NHPs (Fig 4F). Impressive levels of decoy protein were present in nasal ASF with concentrations only slightly reduced in comparison to those achieved with the higher dose.

## Discussion

The rapid emergence of more dangerous and transmissible variants of SARS-CoV-2 in this pandemic is troubling, but not unexpected. The immunological pressures on the virus during natural infections, following antibody therapies, and active vaccines have promoted the emergence of variants[1]. It appears that SARS-CoV-2 improved fitness through mutations that both increased affinity for ACE2 and decreased neutralization by antibodies elicited to precursor strains of the virus[16,26,27]. The density of ACE2 in the nose and airway has been linked to pathogenicity and transmissibility of SARS-CoV-2[28]. The lower levels of ACE2 in the proximal airways of children may be responsible for the lower infection rates and milder symptoms in this group[29]. It has been proposed that SARS-CoV-2 variants achieve greater infection and transmission through increased affinity[26,30]; here we confirm increased affinity for ACE2 in SARS-CoV-2 strains under positive selection during 2020.

Our original goal in engineering the ACE2 decoy was to improve its potency against SARS-CoV-2, which we accomplished through affinity maturation against the Wuhan-Hu-1 spike protein in a yeast display system. Our selection strategy yielded a decoy with high binding and/or neutralizing activity against a full range of SARS-CoV-2 variants, including B1.1.7/alpha, B1.351/beta, B.1.617.1/kappa, and P.1/gamma, which emerged from the UK, the Republic of South Africa, India, and Brazil respectively. However, we were surprised to see equally potent binding and neutralization against other sarbecoviruses, including SARS-CoV-1, which was responsible for the 2003 SARS pandemic. The presence of several second-shell mutations in the affinity-matured decoy may contribute to this breadth since the majority of the ACE2 contact surface was preserved. The engineered decoy does feature some substitutions in direct contact with CoV-2-RBD, which may limit compatibility with some CoVs; further tests against a wider variety of CoV-2 variants as well as a larger panel of ACE2-dependent CoV sequences will be required to fully define the limits of CDY14HL-Fc4 activity on variants fit to infect ACE2-expressing cells. However, the evidence collected so far is consistent with the theory that our ACE2 decoy activity is likely to be enhanced by further CoV-2 viral evolution, since the primary driver of fitness–higher binding to its receptor–has so far mostly enhanced the potency of the decoy (Table 1).

We focused on intranasal delivery of AAV to express CDY14HL-Fc4 to prevent COVID-19. We used the previously described hACE2-TG mouse challenge model to demonstrate efficacy of the decoy *in vivo*. Treated animals lost less weight, showed reduced lung pathology, and showed less replication of the challenge virus. Our results are consistent with the use of this model to evaluate convalescent plasma[23], protease inhibitors[31], and monoclonal antibodies[32], where weight loss, pulmonary pathology, and viral load were decreased, but not completely abrogated.[23] We believe that the mouse challenge model underestimates the potential efficacy of IN AAV-CDY14HL-Fc4. The dose of SARS-CoV-2 that results in human infection is likely much lower, and therefore, easier to neutralize than the inoculating dose used in the mouse challenge model (2.5x10^6^ particles or 280 PFU). We used a novel AAV Clade A capsid called AAVrh91 to maximize transduction in the proximal airways of NHPs. At a relatively low dose (5x10^11^ GC), we achieved levels of CDY14HL-Fc4 in the ASF that should be sufficient to neutralize SARS-CoV-2 variants. Based on our previous studies—using AAV to deliver broadly neutralizing antibodies against influenza—we believe expression should be durable for at least six months and can be effectively administered in the presence of neutralizing antibodies to the vector capsid as a result of a natural AAV infection or a previous administration of this or another AAV gene therapy product[4–7,33]. Further studies will determine if a capsid antibody response to this product will interfere with an AAV treatment for another indication, although we think this risk will be low due to the small quantity of vector delivered and, if present, will be limited to use of a similar capsid in indications where low dose AAV is injected into the blood [34]. An immune response against the engineered decoy transgene in humans is also possible and could impact safety and efficacy of the prophylaxis. However, anti-transgene immune responses in human gene therapy have been documented in only a few instances, mostly in the context of intramuscular route of administration [34]. Additionally, evidence also suggests that secreted transgenes may pose less risk for immunogenicity [34]. We intend to evaluate anti-drug antibodies in future ACE2 decoy studies and as an ongoing measurement of safety in potential future clinical trials.

The emergence of three lethal and highly contagious CoV outbreaks in two decades–SARS in 2003, MERS in 2012, and COVID-19 in 2019 –suggests that CoVs will remain a threat to global health. Surveillance of potential zoonotic sources of these CoVs, such as bats, revealed reservoirs of related viruses capable of evolution and cross-species transmission[35]. One possible therapeutic application of CDY14HL-Fc4 is in the prevention and treatment of future outbreaks caused by new CoVs that utilize ACE2 as a receptor. GTP404 could be rapidly deployed from stockpiles to contain the initial outbreak and the CDY14HL-Fc4 or -Fc1 protein can be leveraged to improve outcomes in those who are infected. The CDY14HL products may be useful in the current COVID-19 pandemic if SARS-CoV-2 variants confound current treatment and prevention strategies. An immediate application could be in immune-suppressed individuals who do not respond to traditional vaccines, develop chronic infection with SARS-CoV-2, and may be reservoirs for new variants[1]. The advantage of vector-expressed decoy in preventing COVID-19 infections in immune-suppressed individuals is that this therapy does not rely on the recipient’s adaptive immune system to be effective.

## Materials and methods

### Ethics statement for study conducted at BIOQUAL (hACE2 TG mouse challenge study)

This research was conducted under BIOQUAL Institute Institutional Animal Care and Use Committee (IACUC) approved protocol number 21–005, in compliance with the Animal Welfare Act and other federal statutes, and regulations relating to animals and experiments involving animals. BIOQUAL is accredited by the Association for Assessment and Accreditation of Laboratory Animal Care International and adheres to principles stated in the Guide for the Care and Use of Laboratory Animals, National Research Council. Animals were monitored twice daily for clinical signs (specifically ruffled fur, heavy breathing, lethargy) and weighed daily.

### Yeast display

We generated mutagenized ACE2 gene fragments by error prone PCR using the Diversify PCR Random Mutagenesis Kit (TakaraBio) at multiple mutation levels, mixing the PCR products. We used Gap-repair cloning and high-efficiency LiAc transformation[36] to assemble the ACE2 gene fragments into a centromeric plasmid. The plasmid contained an upstream Aga2 gene fragment, a downstream HA epitope tag with flexible GSG linkers, and was driven by an inducible GAL1 promoter, and contained a low-copy centromeric origin, similar to pTCON2[37]. Following transformation or sorting rounds, we passaged the libraries at 10X diversity 3 times in SD-trp before inducing in log phase for 24 hrs at 30°C in SG-CAA[37]. For FACS, we stained the yeast with recombinant CoV2 RBD-His6 (Genscript) at diminishing concentrations through the rounds (5nM, 1nM, 0.1nM +/- extended washes of up to 17 hrs for off-rate sorting). We followed up with Mouse anti His6 (Genscript), rabbit anti-HA-PE (Cell Signaling Technology), and goat anti mouse-488 secondary antibody (ThermoFisher) all in phosphate buffered saline (PBS) with 0.1% BSA. Libraries were sorted and analyzed for RBD binding and ACE2 expression at the Penn Flow Core on BD Influx and BD FACSAria II instruments. We extracted plasmid from clones or pools of clones using the Yeast Plasmid MiniPrep Kit (Zymo) and transformed this into bacteria for amplification.

### Next-Generation sequencing and analysis

We performed 2x250 paired-end Illumina sequencing on randomly sheared and size-selected ACE2 amplicons from the yeast display rounds. After removing adapters and low-quality reads, we mapped clean reads no shorter than 200bp to the WT ACE2 nucleotide sequences using NovoAlign (v.4.03.01). We translated in-frame sequences with mapping quality scores no lower than 30 and without indels into amino acid sequences and compared to the WT ACE2 protein sequence. We tallied non-synonymous changes for each codon across the ACE2 sequence (18–615). We calculated mutation rates at each codon as follows: (sum of non-synonymous AAs)/(sum of all AAs).

### Expression of ACE2 variant IgG Fc4 fusions and RBDs

We sub-cloned candidate ACE2 synthetic DNA or decoy sequences from the yeast display format into pCDNA3.1, using the endogenous ACE2 signal peptide and appending a human IgG4 Fc domain (residues 99 to 327 from Uniprot reference sequence P01861) and a C-terminal His6 tag. To generate protein for screening we transiently transfected HEK293 cells with plasmid DNA in six-well plates using PEI and collected and clarified supernatant 72 hours later. We quantified expression using the IgG4 Human ELISA kit (Invitrogen BMS2095) with IgG4 standards provided in the kit. For CDY14-Fc4 and CDY14HL-Fc4, we produced the protein in a similar manner but purified it on protein A sepharose followed by size-exclusion chromatography on Superose 6 resin (Cytiva) to remove inactive aggregates. We determined the concentration using the predicted extinction coefficient at 280nm (1 g/l = 1.995). Some neutralization measurements (e.g., S3 Fig) used crude decoy protein titered using a human IgG4 ELISA kit; this typically yielded higher IC50s. We cloned the synthetic sequences (IDT gBlocks) of RBD [Spike amino acids 330–530 (CoV2), 317–516 (CoV1), and 318–517 (WIV1-CoV)] into pCDNA3.1 between an IL2 signal peptide plus Gly-Ser and a C-terminal His6 tag. We transfected RBD plasmids into HEK293 cells using PEI and collected supernatants 72 hrs later for clarification, concentration, and purification on Ni-NTA resin, followed by dialysis into PBS. We confirmed purity using Coomassie-stained SDS-PAGE analysis. We determined concentrations of the RBD using a predicted extinction coefficient at 280nm.

### RBD binding with SPR

We performed SPR binding analysis using a Biacore T200 instrument (GE Healthcare) at room temperature in HBS-EP(+) buffer (10 mM HEPES pH 7.4, 150 mM NaCl, 3 mM EDTA, and 0.05% P20 surfactant, Cat# BR100669, Cytiva) using a protein A/G derivatized sensor chip (Cat# SCBS PAGHC30M, XanTec Bioanalytics). We injected WT ACE2-hFc4 or CDY14-hFc4 diluted to 60nM in HBS-EP(+) at a flow rate of 10 μL/min for 3 min to capture ~1,000 response units (RU) on the sensor surface in each cycle. We measured binding of various SARS-CoV RBD proteins to this surface at concentrations ranging from 200 nM to 0.195 nM. RBD binding was measured at a flow rate of 30 μL/min, with a 3 min association time and a 15 min dissociation time. We performed regeneration between binding cycles using 10 mM glycine pH 1.5 injected at a flow rate of 60 μL/min for 1 min. KD values were determined for each interaction using kinetics parameter fitting in the Biacore T200 Evaluation software. We used a global 1:1 binding model and did not adjust for refractive index shift. Data presented are the average of two or more replicates measured for each RBD domain tested.

### CoV pseudotyped lentiviral neutralization assay

We obtained non-replicating lentivirus pseudotyped with CoV spike proteins from Integral Molecular [catalog numbers 701, 702, 704, 706, 707, 708, 710, 712, 713, 711, 726, and Urbani-Cov1]. The reporter virus particles encoded a Renilla luciferase reporter gene. We set up neutralization reactions with 100 ul of inhibitor diluted in full serum media and 10 ul of reporter virus. After 1 hour at 37°C, we added 20,000 cells/well in 50 ul of a HEK 293T cell line overexpressing ACE2 (Integral Molecular) and incubated the cells for 48 hours. We measured reporter virus transduction activity on a luminometer (BioTek) using the Renilla Glo Kit (Promega) following manufacturer’s instructions. For higher throughput screens of neutralizing potency, we used crude expression supernatant (described above) in the neutralization assay at 1 or 2 dilutions (typically 10- or 100-fold). We transformed the luciferase reading to an estimated potency (EP) using the following formula: EP = (L*[decoy])/(1-L), where L is the fractional luciferase level as compared to a mock sample (no inhibitor), and [decoy] is the concentration of the decoy in the neutralization well as titered using a human IgG4 ELISA kit. This was sufficient to rank clones without performing a full titration.

### AAV vector production

The University of Pennsylvania Vector Core produced recombinant AAV vectors as previously described [38,39].

### Decoy quantification by mass spectrometry

#### Standards

Soluble hACE2Fc (produced in-house) was spiked at different levels (0.5–500 ng/mL) into PBS or NLF acquired from a naïve rhesus macaque. Samples were denatured and reduced at 90°C for 10 minutes in the presence of 10mM dithiothreitol (DTT) and 2M Guanadinium-HCl (Gnd-HCl). We cooled the samples to room temperature, then alkylated samples with 30mM iodoacetamide (IAM) at room temperature for 30 minutes in the dark. The alkylation reaction was quenched by adding 1μL DTT. We added 20mM ammonium bicarbonate to the denatured protein solution, pH 7.5–8 at a volume to dilute the final Gnd-HCl concentration to 200mM. Trypsin solution was added at ~4ng of trypsin per sample ratio and incubated at 37°C overnight. After digestion, formic acid was added to a final concentration of 0.5% to quench the digestion reaction.

#### LC–MS/MS

We performed online chromatography with an Acclaim PepMap column (15 cm long, 300-μm inner diameter) and a Thermo UltiMate 3000 RSLC system (Thermo Fisher Scientific) coupled to a Q Exactive HF with a NanoFlex source (Thermo Fisher Scientific). During online analysis, the column temperature was regulated to a temperature of 35°C. Peptides were separated with a gradient of mobile phase A (MilliQ water with 0.1% formic acid) and mobile phase B (acetonitrile with 0.1% formic acid). We ran the gradient from 4% B to 6% B over 15 min, then to 10% B for 25 min (40 minutes total), then to 30% B for 46 min (86 minutes total). Samples were loaded directly to the column. The column size was 75 cm x 15 um I.D. and was packed with 2-micron C18 media (Acclaim PepMap). Due to the loading, lead-in, and washing steps, the total time for an LC-MS/MS run was about 2 hours.

We acquired MS data using a data-dependent top-20 method for the Q Exactive HF; we dynamically chose the most abundant not-yet-sequenced precursor ions from the survey scans (200–2000 m/z). Sequencing was performed via higher energy collisional dissociation fragmentation with a target value of 1e5 ions, determined with predictive automatic gain control. We performed an isolation of precursors with a window of 4 m/z. Survey scans were acquired at a resolution of 120,000 at *m*/*z* 200. Resolution for HCD spectra was set to 30,000 at *m*/*z*200 with a maximum ion injection time of 50 ms and a normalized collision energy of 30. We set the S-lens RF level at 50, which gave optimal transmission of the *m*/*z* region occupied by the peptides from our digest. We excluded precursor ions with single, unassigned, or six and higher charge states from fragmentation selection.

#### Data processing

We used BioPharma Finder 1.0 software (Thermo Fischer Scientific) to analyze all data. For peptide mapping, we used a single-entry protein FASTA database to perform searches. The mass area of the target peptide was plotted against the spike concentration to complete a standard curve.

#### Selection of target peptide

Based on initial *in silico* studies, we selected four peptides as possible sequence-specific matches for targeted quantification. We evaluated sensitivity performance for quantification of the four peptide targets in the NLF background matrix. Following blank injections to establish system cleanliness, replicate injections (n = 3) were made at all levels, from 0.5 ng/mL to 500 ng/mL. Three of the peptides were detected with ANHYEDYGDYWR providing the greatest response across the whole range. We determined retention time (RT) reproducibility across all samples (n = 24) and determined peak area reproducibility and quantification accuracy for each level. Excellent linearity was observed for the levels tested with typical R^2^ > 0.94 for ANHYEDYGDYWR. For ANHYEDYGDYWR, we observed excellent precision and accuracy at all levels, with all replicates within 10% CV. For test articles, 1x or 10x NLF and/or bronchoalveolar lavage fluid (BAL) was treated as previously described without any dilution or protein precipitation. The mass area of target peptide in test articles was compared to the linear calibration generated for the spiked material to determine the level of decoy present in the test article.

### ASF dilutions from serum and lavage urea

We used the urea concentrations in BAL or NLF and in serum collected at the same time to determine the dilution that the lavage introduced to the ASF[40]. We quantified urea in mouse BAL and serum, and in NHP NLF using the Urea Assay Kit (Abcam). We obtained serum urea concentrations from NHP from the blood urea nitrogen as part of standard bloodwork lab panels (Antech).

### Spike binding ELISA

SARS-CoV-2 Spike Protein RBD (Sinobio #40592-V08H) was immobilized on a 96-well plate (0.25ug/mL in PBS, 100ul/well) at 4°C overnight. Plates were then washed 5x with PBS/0.05% Tween and blocked with PBS/1.0% BSA for 1 hour with shaking. Samples (2x dilution in PBS/2.0% BSA) and standards (soluble hACE2-Fc at starting concentration 100ng/ml, 12-point, 1:2 serial dilution, plus a 0.0ng/ml blank, in PBS/1.0% BSA) were added at 100ul/well in duplicate and incubated for 2 hours at room temperature with shaking. Wells were washed as described and biotin-conjugated goat anti-human IgG (Jackson AffiniPure #109-065-098; 1:30,000 or Southern Biotech 2049–08; 1:1,000) in PBS/1.0% BSA detection antibody was added to the wells at 100ul/well and incubated for 2 hours at room temperature with shaking. Wells were washed as described, followed by the addition of 100ul/well Streptavidin-HRP (Abcam #ab7403; 1:30,000) in PBS/1.0% BSA for 30 minutes with shaking. Wells were washed as described and incubated in 100ul/well TMB substrate (Seracare #5120–0076) in the dark at room temperature with shaking until the reaction was stopped with 100ul/well TMB Stop Solution (Seracare #5150–0021). Absorbances were read at 450 nm using a Spectramax M3 plate reader. We exported and analyzed the data in GraphPad Prism Version 9.0.2. All raw data were blank subtracted. We plotted a standard curve of soluble hACE2-Fc, and the X-axis (concentration) was log_10_ transformed. We performed a 4-parameter nonlinear regression upon the transformed standard curve, and interpolated sample concentrations.

### Determination of Matrix Interference in BAL and NLF samples

Soluble hACE2Fc was spiked into NLF (0.0, 0.5, 2.0, and 10.0 ng/ml) acquired from a naïve rhesus macaque on the same plate with a standard curve (soluble hACE2Fc starting concentration 100ng/ml, 12-point, 1:2 serial dilution, plus a 0.0ng/ml blank) in PBS/1.0% BSA. We performed the spike binding assay and data analysis as described above.

### Expression study in mice

All animal procedures were performed in accordance with protocols approved by the Institutional Animal Care and Use Committee of the University of Pennsylvania. C57BL/6J mice were purchased from The Jackson Laboratory. Anesthetized mice received an IN administration of 10^11^ GC of AAVhu68.CDY14-Fc4, AAVrh91.CDY14-Fc4, AAVhu68.CDY14HL-Fc4, or AAVrh91.CDY14HL-Fc in a volume of 50 μL or the same volume of vehicle control (PBS) on day 0. On day 7, mice were euthanized and BAL was collected (1 ml of PBS administered intratracheally).

### hACE2 TG mouse study

To evaluate the prophylactic efficacy potential of AAV expressing hACE2 receptor decoys against SARS-CoV-2, BIOQUAL, Inc. (Rockville, MD) conducted a challenge study using hACE2 TG mice (Stock No: 034860, The Jackson Laboratory). Mice were administered with either vehicle or 10^11^ GC of AAVhu68.CDY14HL-Fc4 IN on day -7 as described above. On day 0, mice were administered with mock or the SARS-CoV-2 challenge (50 μl of 2.8x10^2^ pfu of SARS-CoV-2, USA_WA1/2020 isolate [NR-52281, BEI Resources]). Mice were euthanized on either day 4 or 7 via cervical dislocation. BAL was collected as described above and aliquoted for viral load assays into Trizol LS (Thermo Fisher Scientific, Waltham, MA) or heat inactivated (60°C for 30 minutes) for decoy protein expression. The lung was collected and split for histopathology into 10% neutral buffered formalin or snap frozen for viral load analysis. RNA extraction for RT-qPCR, the quantitative RT-PCR assay for SARS-CoV-2 RNA, and subgenomic RNA were performed as described[41].

### Histopathology of collected organs

The organs collected at necropsy were trimmed and routinely processed for hematoxylin and eosin (H&E) staining. Slides were blindly evaluated by a blinded pathologist using a severity score of 0 (no lesions observed), 1 (minimal), 2 (mild), 3 (moderate), 4 (marked) and 5 (severe) for each finding.

### Intranasal capsid comparison by AAV barcoding

We generated a set of custom barcoded plasmids using degenerate nucleotides that anneal immediately downstream of the stop codon in a GFP reporter construct that contains the AAV2 ITRs. We produced barcoded AAV vectors for each serotype in the study separately by transfecting HEK293 cells as described[38], replacing the typical single ITR-containing plasmid in the transfection mix with an equimolar mixture of 4 uniquely barcoded reporter constructs. We pooled the individual vector preps on an equimolar basis using their digital droplet PCR titers. We determined the absolute barcode distribution in the AAV pool by deep sequencing; we extracted AAV genomes from the pool and performed linear-range PCR using primers that flank the barcode region to generate an amplicon for paired-end Illumina sequencing.

### NHP studies

Rhesus and cynomolgus macaques were obtained from Primgen (PreLabs). NHP studies were conducted at the University of Pennsylvania or Children’s Hospital of Philadelphia within facilities that are United States Department of Agriculture-registered, Association for Assessment and Accreditation of Laboratory Animal Care-accredited, and Public Health Service-assured. For the barcode study, 4x10^12^ GC of the pool AAV preps was delivered IN in a total volume of 0.28 ml to an adult male rhesus macaque using the MAD Nasal device. After 14 days, we collected airway tissues at necropsy, and extracted total RNA using Trizol Reagent (Thermo Fisher). We generated cDNAs using Superscript III reverse transcriptase (ThermoFisher) and an oligo dT primer. We used the cDNAs to prepare barcode amplicons for Illumina sequencing as described above. We extracted the relative barcode abundances in input (AAV mixture) and output (tissue cDNAs) from Illumina data. The ratio of output to input relative abundances (“mRNA barcode enrichment”, Fig 4C and 4D) for each barcode in each tissue is proportional to the relative efficiency of the capsid linked to that barcode in that tissue. Agreement among the 4 barcodes assigned to each capsid allows us to assess assay noise and detect rare, tissue-specific effects of the barcode itself on transcript stability (none detected). For each tissue, we quantified the total capsid-derived transcript per ug of total RNA using qPCR with a primer/probe set common to all the barcoded reporters. Using the total transcript counts and capsid-specific barcode distribution from NGS of the cDNA barcode amplicon allowed us to quantify absolute transduction from each capsid in a particular tissue (Fig 4E).

For the decoy expression in NHPs, cynomolgus macaques (n = 2/vector) were administered IN with 5x10^12^ GC of AAVhu68.CDY14, AAVrh91.CDY14, AAVhu68.CDY14HL, or AAVrh91.CDY14HL as described above. An additional two NHPs were administered with 5x10^11^ GC of AAVrh91.CDY14HL. All NHPs were negative for pre-existing neutralizing antibody titers to the administered AAV capsid prior to study initiation (Immunology Core at the Gene Therapy Program). Animals were monitored throughout the in-life phase for complete blood counts, clinical chemistries, and coagulation panels by Antech Diagnostics (Lake Success, NY). On days 7, 14, and 28 NLF was collected (animals placed in ventral recumbency with head tilted to the right, up to 5 ml of PBS delivered in 1ml aliquots, and fluid collected via gravity). Animals were necropsied on day 28 and a full histopathological evaluation was performed.

### Statistical analysis

Statistical analyses performed using *R* (version 4.0.0). Statistical tests described in figure legends.

## Supporting information

S1 Fig Design and characterization of initial ACE2 decoy construct. **A. Schematic representation of the initial ACE2-Fc4 decoy constructs.** ACE2 decoy constructs contained the extracellular domain of ACE2 (amino acids 18–615) with one of three candidate signal peptides (IL-2, native, or thrombin). In some constructs, two catalytic histidine residues were mutated to asparagine to abrogate enzymatic activity (designated NN). Constructs were designed with no Fc, or the Fc domain of IgG1 or IgG4. B. Constructs were expressed in HEK293 cells and detected in supernatant using a sandwich ELISA to ACE2 (for constructs without an Fc domain) or an ELISA with SARS-CoV-2 spike protein as a capture antigen and an anti-human IgG polyclonal antibody for detection (for Fc fusion proteins). C. The candidate ACE2-NN-Fc4 fusion protein was expressed in HEK293 cells, purified by protein A chromatography, and analyzed by SDS PAGE under reducing and nonreducing conditions D. The affinity of the purified ACE2-NN-Fc4 decoy protein for monomeric spike protein in solution was quantified by Biacore SPR. kon = 2.6 x 105 M-1 s-1, koff = 0.00093 s-1, t1/2 = 745 s, KD = 3.5 nM, Rmax = 67 RU. E. The purified ACE2-NN-Fc4 protein was titrated against Wuhan CoV2 pseudotyped lentivirus bearing a luciferase reporter. The IC50 was obtained from a fit of these data (15 ug/ml) F. The candidate construct (ACE2-NN-Fc4) was packaged in an AAV vector (hu68 capsid) and administered IN to WT mice. Seven days after administration, BAL was collected for measurement of transgene expression using an ELISA with SARS-CoV-2 spike protein as a capture antigen to confirm that the decoy receptor expressed *in vivo* was functional. BAL from similar experiments was 6-fold diluted from the ASF as determined by comparison of BAL and serum urea. Thus, we determined that ASF concentrations of the decoy were likely below 2 ug/ml. G. Two NHPs (IDs 258 and 396) received 9 x 10^12^ GC of an AAVhu68 vector expressing a soluble ACE2-NN-Fc fusion protein via the MAD (Fig 4). Nasal lavage samples were collected weekly after vector administration and concentrated 10-fold for analysis. The concentration of the decoy receptor in NLF was measured by MS. Urea measurements in similar experiments indicate that 10X nasal lavage is ~8-fold diluted from ASF. We therefore determined that ASF concentrations of the decoy were less than 100 ng/ml.
(TIF)

S2 Fig Design and selection of primary and secondary yeast display libraries. A. Structure of CoV-2 RBD (blue spheres) bound to human ACE2 (green ribbons, red and yellow spheres) (6M17.pdb (41)). Most ACE2 contacts with RBD are limited to the amino acids 18–88 (red spheres) and a patch of amino acids that are more C terminal (yellow spheres). The gene sequence for ACE2 is shown below in the same coloring. B. We designed two primary yeast display libraries: 1) the whole ACE2 gene fragment was mutagenized (Whole) and 2) the mutagenesis was limited to only the first 96 amino acids (NC) to concentrate the mutagenesis on the region most likely to impact RBD binding. The regions shaded gray were subjected to error-prone PCR to introduce mutations. C. Deep sequencing of yeast display plasmids extracted from the final round of sorting for the Whole and NC libraries. The fractional rate of mutation at each position in 18–615 of ACE2 is plotted. Improving mutations occurred mostly in the first 96 amino acids regardless of the input library. These include RBD contact residues, second-shell residues, and the distal consensus N-glycan site at position 90, an apparent negative regulator of RBD binding. Several consensus C-terminal mutations emerged from the whole ACE2 primary sorts. These include a substitution to Y at position 330, which we identified in a clone with improved binding. D. A detailed plot of the mutational frequencies in Whole and NC library final round sorts for residues 18–100. The libraries yielded many of the same mutants in this region with improved binding activity. E. Schematic representation of secondary library design. We isolated 300 yeast colonies from the sorted primary (Whole and NC) libraries, analyzed them individually for RBD binding and ACE2 expression by flow cytometry, and selected 90 isolates with validated binding improvements. Next we generated a secondary library by shuffling selected ACE2 genes using the staggered extension process (StEP) method(9). Given that most improving mutations were N-terminal, we shuffled only residues 18–103 of the input templates (orange shaded region in the schematic), matching these with a mixture of unmutated and N330Y C-terminal DNAs in a multi-fragment assembly yeast transformation.
(TIF)

S3 Fig Alternative decoy engineering strategy and decoy candidate characterization by neutralization assays. A. Schematic of parallel paths to the generation of affinity matured ACE2 decoy. After generating improved RBD binding sequences from a primary round of sorting, we undertook a parallel path to digitally recombine the most frequent mutations in addition to continued diversification and sorting. Primary library hits, digital recombinants of those hits and isolated clones from the second, more stringent round of yeast display sorting were all cloned as Fc4-fusion proteins and screened in a CoV2 pseudotype neutralization assay. B. We selected 300 clones from primary yeast display library sorts for clonal RBD binding analysis using flow cytometry in the yeast display format and selected 90 clones with validated binding improvements. A pool of plasmid DNA from those 90 isolates was subjected to deep sequencing for mutational analysis, and the rates of all possible amino acid substitutions are presented in this heat map by amino acid position. Black squares represent the wt amino acid at each position. C. The goal was to generate a collection of synthetic (digital) recombinants of the observed mutations in this data set. We grouped subsets of the validated mutations into 7 regions (p1 -p7) and assigned frequencies to the mutations based loosely on observed frequencies in the data set, allowing for the wt residue at 10% or 50% depending on the position. We biased the mutation selection towards second-shell positions to avoid directly remodeling the ACE2:RBD contact positions where possible. D. In order to maximize the chance of mutations working together productively, we chose the groupings in (C) based on 3D structure (6M17.pdb (40)) such that direct contacts between groups would be minimized. We randomly selected primary screen mutation combinations based on the frequencies in (C), and had these digital recombinants synthesized for cloning as secreted IgG Fc4 fusion proteins. E. We screened primary yeast display hits, digital recombinants, and secondary yeast display hits in a pseudotyped lentivirus reporter assay for CoV-2 neutralization at one or two dilutions from the expression supernatant, noting the expression titer relative to ACE2-wt-Fc4 control. F. Mutations associated with the 5 best digital recombinant hits and the 5 best hits from a secondary round of yeast display sorting. G. Titration of Wuhan CoV-2 pseudotyped reporter lentivirus with CDY14HL-Fc4 (with protease inactivating H345L) or CDY14-Fc4 (active site intact). The decoy proteins used in (G) were protein A purified, but likely contained some inactive aggregates that reduced their specific activities.
(TIF)

S4 Fig Example SPR data for RBD binding assay. A. Raw data (colored lines) and fits (black lines) for immobilized ACE2-wt-Fc4 on the SPR chip surface binding injected RBDs (concentrations listed). Parameters of the fits, including the dissociation equilibrium constant (KD) are listed below each panel. These data contributed to Fig 2B and 2C. B. Raw data (colored lines) and fits (black lines) for immobilized CDY14HL-Fc44 on the SPR chip surface binding injected RBDs (concentrations listed).
(TIF)

S5 Fig Challenge study in mice. Average weight loss (percentage) in males A. and females B. hACE2-TG mice that received Challenge Placebo and Challenge Decoy.
(TIF)

S6 Fig AAV capsid selection for NHP IN delivery. A-F. mRNA barcode enrichment in airway tissues for a mixture of 9 barcoded serotypes delivered IN at 2.7E11 GC each. We assigned four uniquely barcoded transgenes to each capsid at manufacture. Data show the enrichment score (tissue abundance in RT-PCR-NGS/ injection mixture abundance in PCR-NGS) for all 4 barcodes per capsid with mean and SD. H. AAV construct design for pilot studies with CDY14HL-Fc4 and CDY14-Fc4. I and J. Four NHP were IN dosed with rh91 or hu68 vectors encoding decoy transgenes at 5E12 GC. Data show the biodistribution of vector genomes in airway tissues 28 days after dosing. Rh91 achieved higher gene transfer in upper airway tissues (I), particularly in the maxillary sinuses and cavity septum. Gene transfer in lower airway tissues was more variable (J).
(TIF)
